# Effects of Zinc, Copper and Iron Oxide Nanoparticles on Induced DNA Methylation, Genomic Instability and LTR Retrotransposon Polymorphism in Wheat (*Triticum aestivum* L.)

**DOI:** 10.3390/plants11172193

**Published:** 2022-08-24

**Authors:** Kamil Haliloğlu, Aras Türkoğlu, Özge Balpınar, Hayrunnisa Nadaroğlu, Azize Alaylı, Peter Poczai

**Affiliations:** 1 Department of Field Crops, Faculty of Agriculture, Ataturk University, Erzurum 25240, Turkey; 2 Department of Biology, Faculty of Science, Cankiri Karatekin University, Cankırı 18200, Turkey; 3 Department of Field Crops, Faculty of Agriculture, Necmettin Erbakan University, Konya 42310, Turkey; 4 Hemp Research Institute, Ondokuz Mayıs University, Samsun 55200, Turkey; 5 Department of Food Technology, Vocational College of Technical Sciences, Ataturk University, Erzurum 25240, Turkey; 6 Department of Nano-Science and Nano-Engineering, Institute of Science, Ataturk University, Erzurum 25240, Turkey; 7 Department of Nursing, Faculty of Health Sciences, Sakarya University of Applied Sciences, Sakarya 54187, Turkey; 8 Botany Unit, Finnish Museum of Natural History, University of Helsinki, P.O. Box 7, FI-00014 Helsinki, Finland; 9 Institute of Advanced Studies Kőszeg (iASK), P.O. Box 4, H-9731 Kőszeg, Hungary

**Keywords:** DNA methylation, genomic instability, in vitro, nanoparticles, retrotransposons, wheat

## Abstract

Nanomaterials with unique and diverse physico-chemical properties are used in plant science since they improve plant growth and development and offer protection against biotic and abiotic stressors. Previous studies have explored the effects of such nanomaterials on different plant mechanisms, but information about the effects of nanomaterials on induced DNA methylation, genomic instability and LTR retrotransposon polymorphism in wheat is lacking. Therefore, the present study highlights the key role of nanoparticles in DNA methylation and polymorphism in wheat by investigating the effects of ZnO, CuO and γ-Fe_3_O_4_ nanoparticles (NPs) on mature embryo cultures of wheat (*Triticum aestivum* L.). Nanoparticles were supplemented with Murashige and Skoog (MS) basal medium at normal (1X), double (2X) and triple (3X) concentrations. The findings revealed different responses to the polymorphism rate depending on the nanoparticle type and concentration. Genomic template stability (GTS) values were used to compare the changes encountered in iPBS profiles. ZnO, CuO and γ-Fe_3_O_4_ NPs increased the polymorphism rate and cytosine methylation compared to the positive control while reducing GTS values. Moreover, non-γ-Fe_3_O_4_ NPs treatments and 2X ZnO and CuO NP treatments yielded higher polymorphism percentages in both *Msp*I- and *Hpa*II-digested CRED-iPBS assays and were thus classified as hypermethylation when the average polymorphism percentage for *Msp*I digestion was considered. On the other hand, the 3X concentrations of all nanoparticles decreased *HpaII* and *MspI* polymorphism percentages and were thus classified as hypomethylation. The findings revealed that MS medium supplemented with nanoparticles had epigenetic and genotoxic effects.

## 1. Introduction

Materials with sizes of 1–100 nanometers are known as nanomaterials. The supplementation of such nanomaterials into different substances such as fertilizers and plant nutrients may improve the quality-related traits of these substances. Indeed, studies have revealed the effects of nanoparticle-containing nutrients on plant growth and development [1,2]. Since nano-sized structures are more effective than bulk materials, nutrients containing nanomaterials may have much more toxic or beneficial effects on plants [3]. For example, Zhang et al. [4] investigated the effects of many nanoparticle structures, including gold (Au), copper (Cu), Zinc (Zn), aluminum (Al), titanium oxide (TiO_2_), zinc oxide (ZnO), copper oxide (CuO) and silver (Ag), on plants. However, previous studies mainly focused on product development, protection and fertilization. 

Wheat (*Triticum aestivum* L.) has an essential role in world nutrition. Notably, stress factors significantly influence wheat plants; serious quality and yield losses are encountered under stress conditions. As such, biotic and abiotic stressors result in changes in wheat DNA [5,6]. Nanotechnological applications may have either stress-related negative effects or very effective beneficial effects on wheat yield. Therefore, it is thought that nanotechnology has tremendous potential to positively affect yield factors [7]. Accordingly, the effects of nanoparticles on plants have been the primary focus of many research projects in recent years. According to [8,9], nanoparticles enter plant cells and leaves and can also transport DNA and chemicals into plant cells. The majority of studies on NPs to date concern toxicity. Recently, several studies have shown that heavy metals, such as Cd, Pb, Co, Ni and Zn, cause changes in DNA methylation [10]. However, less is known about the effects of their respective nanoparticles on the induction of specific gene mutations in plants and DNA methylation changes. It is crucial to determine the effect of nanomaterials on plant genetic stability, especially in this period when the use of nanomaterials in plant fertilizers is becoming more and more widespread [11]. Hence, the current study is among the first to explore the alteration of DNA methylation caused by nanoparticles in plants.

By expressing stress-related genes, plants express their responses to environmental stimuli and stress factors. Another parameter as effective as transcription factors in regulating gene expression is epigenetics, which refers to “heritable and reversible changes in gene expression without alterations of underlying DNA sequence” [12]. Gene expression is regulated by three epigenetic mechanisms: DNA methylation, histone modification and miRNA. Observing epigenetic changes can control many vital processes in plants, from growth development to flowering. Therefore, DNA methylation has become one of the most popular epigenetic phenomena [13,14]. 

DNA hyper/hypomethylation is largely correlated with gene expression, cell differentiation and phylogenetic development. DNA methylation is also correlated with several biological processes, including the transcriptional silencing of genes and transposable inactivation of transposons [15]. DNA methylation, microRNA (miRNA) and retrotransposon activities may also alter gene expression profiles and ultimately result in genomic instability [10]. Various techniques, including DArTseqMet [16], semiquantitative MSAP [17], methylRAD [18], methyl-seq [19] and a variant of MSAP [19], have recently been used to detect DNA methylation. Methylation-sensitive amplified fragment length polymorphism (metAFLP) is also used to analyze the changes in DNA methylation [20]. Several other techniques or methods, including HPLC, the bisulfites method, methylation-specific PCR (MSP), the sequencing of specific genes, AFLP, MSAP, coupled restriction enzyme digestion–random amplification (CRED-RA) [21] and coupled restriction enzyme digestion–inter-primer binding site (CRED-iPBS) [22], have also been used to detect DNA methylation changes. The CRED-iPBS method employs the methylation-sensitive enzymes *HpaII* and *MspI* to detect methylation changes [23]. 

There are limited studies on nanomaterial-induced epigenetic changes in plant DNA. This study aimed to determine whether there were epigenetic changes in the DNA of wheat that were previously developed in a medium containing nanoparticles [24]. It is thought that this study will contribute to the literature in determining the effects of nanoparticles on DNA methylation changes in wheat. The study basically measured the genomic instability of wheat against nanomaterials. The main purpose of this research was to observe how wheat would respond genetically to the nanotechnological effects that it may be exposed to in the future, due to the increasing use of nanofertilizers. Therefore, the aim of this study was to determine the effects of nanoparticles on methylation and epigenetic changes encountered in wheat DNA using the CRED-iPBS method. It was hypothesized that the high reactivity of nanoparticles could lead to epigenetic changes in wheat DNA.

## 2. Results

### 2.1. Characterization of Nanoparticles

ZnO, CuO and γ-Fe_3_O_4_ nanoparticles synthesized by green synthesis were characterized by SEM, XRD and FTIR. ZnO, CuO and γ-Fe_3_O_4_ nanoparticles were synthesized at 30 °C, 25 °C and 20 °C, respectively. The analyses revealed that ZnO, CuO and γ-Fe_3_O_4_ nanoparticles had varying sizes of 60–80 nm, 5–120 nm and 30–80 nm, respectively. The reason for the broad size ranges of nanoparticles is the adhesion of tiny metallic nanomaterials synthesized by green synthesis. This situation is why small nanoparticles may appear larger [25]. According to the XRD analysis findings, it was determined that CuO nanoparticles were in face-centered cubic form, γ-Fe_3_O_4_ nanoparticles were spherical and ZnO nanoparticles were in hexagonal crystal from Nadaroğlu et al. [26]. The article presents data associated with the characterization (SEM, XRD and FT-IR) of relevant nanomaterials by Nalci et al. [24].

### 2.2. iPBS Analysis

In total, 20 oligonucleotide primers with approximately 50–70% GC content were used to analyze the PCR products of the T. *aestivum* Kirik genome, but only 10 provided specific and stable results. Compared to the PCR products obtained from the control DNA, nanoparticle treatments resulted in apparent changes in the iPBS patterns. 

**Figure 1:**
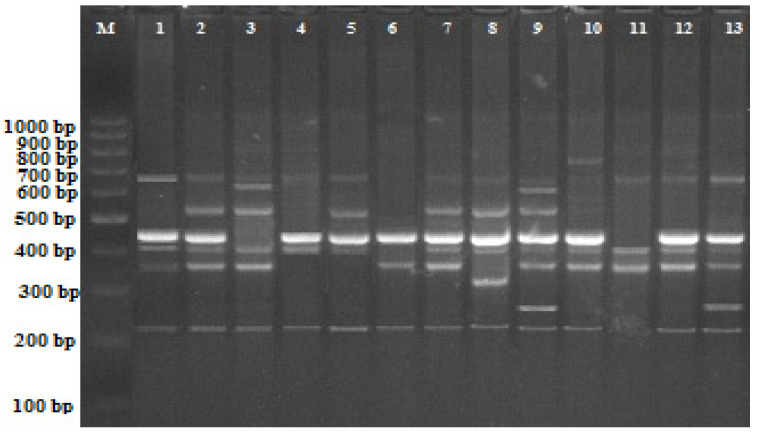
iPBS profiles for various experimental groups with 2391 primers in wheat. M, 100–1000 bp DNA ladder; 1, control; 2, MS medium containing 0X ZnO NPs; 3, MS medium containing 1X ZnO NPs; 4, MS medium containing 2X ZnO NPs; 5, MS medium containing 3X ZnO NPs; 6, MS medium containing 0X CuO NPs; 7, MS medium containing 1X CuO NPs; 8, MS medium containing 2X CuO NPs; 9, MS medium containing 3X CuO NPs; 10, MS medium containing 0X γ-Fe_3_O_4_ NPs; 11, MS medium containing 1X γ-Fe_3_O_4_ NPs; 12, MS medium containing 2X γ-Fe_3_O_4_ NPs; 13, MS medium containing 3X γ-Fe_3_O_4_ NPs.

As shown in Table 1, a total of 67 bands was seen in the control treatment; the highest number of bands was seen in iPBS-2382 (nine bands), while the lowest number of bands was observed in iPBS-2387 and iPBS-2392 (six bands). The molecular sizes of polymorphic bands ranged from 100 (iPBS-2385) to 850 (iPBS-2382) bp. These changes were characterized by a variation in band intensity, the loss of regular bands or the appearance of new bands (as shown in Table 1 and Figure 1). There were significant differences in the iPBS profiles of the control and nanoparticle treatments. Compared to the control, 115 new bands appeared, while 106 bands were not present in the experimental groups. 

Each nanoparticle with different concentrations yielded a different response to the polymorphism rate, and decreasing polymorphism rates were seen with increasing nanoparticle concentrations of the MS medium. Polymorphism rates were respectively measured as 25.37%, 28.35%, 34.32% and 25.37% for 0, 1X, 2X and 3X ZnO treatments; as 26.86%, 23.88%, 29.85% and 22.38 % for 0, 1X, 2X and 3X CuO treatments and as 37.31%, 25.37%, 28.34% and 2.89% for 0, 1X, 2X and 3X γ-Fe_3_O_4_ treatments. 

GTS was used to compare the changes in iPBS profiles. The present findings revealed that ZnO, CuO and γ-Fe_3_O_4_ nanoparticle treatments at different concentrations yielded different responses to GTS values. GTS values were determined respectively as 74.62%, 71.64%, 65.67% and 74.62% for 0, 1X, 2X and 3X ZnO treatments; as 73.13%, 76.11%, 70.14% and 77.61% for 0, 1X, 2X and 3X CuO treatments and as 60.68%, 74.62%, 71.64% and 79.10% for 0, 1X, 2X and 3X γ-Fe_3_O_4_ treatments. The results strongly showed that the application of all nanoparticles with 3X concentration (particularly γ-Fe_3_O_4_ nanoparticle) enhanced GTS values under the tissue culture approach (Table 1).

### 2.3. CRED-iPBS Analysis

Among the 20 iPBS primers, 10 produced specific and stable bands and were used in CRED-iPBS analysis (Table 2). CRED-iPBS analysis enabled the observation of any possible cytosine methylations caused by non-γ-Fe_3_O_4_ treatments. Enhancement was also seen in cytosine methylation with CuO treatments. The results of the CRED-iPBS analysis as the average polymorphism percentages in terms of *Hpa*II and *Msp*I digestions are provided in Table 2. In total, 69 and 67 bands were observed in the *Msp*I- and *Hpa*II-digested control treatments, respectively. According to the results of the CRED-iPBS pattern, the *MspI* polymorphism percentage was higher than the *HpaII* polymorphism percentage in all nanoparticle treatments. There were apparent differences in the *MspI* polymorphism percentages of the experimental groups. Significantly higher polymorphism rates were observed in the control treatments. The present findings revealed that ZnO, CuO and γ-Fe_3_O_4_ nanoparticle treatments at different concentrations yielded different responses for the polymorphism percentages of *MspI* and *HpaII* digestion. 

For *MspI* digestion, polymorphism percentages were respectively determined as 32.77%, 41.34%, 50.88% and 31.50% for 0, 1X, 2X and 3X ZnO treatments, as 35.60%, 29.50%, 43.01% and 24.84% for 0, 1X, 2X and 3X CuO NPs treatments and as 57.96%, 32.28%, 37.94%% and 23.85% for 0, 1X, 2X and 3X γ-Fe_3_O_4_ NPs treatments. There was a clear increase in polymorphism percentage from 0X to 2X in ZnO NPs treatments but a decrease from 2X to 3X in ZnO NPs treatments. With CuO NPs treatments, a decrease was seen in polymorphism percentage from 0X to 1X, an increase from 1X to 2X and a decrease from 2X to 3X. With γ-Fe_3_O_4_ NPs, a decrease was seen in polymorphism percentage from 0X to 1X, an increase from 1X to 2X and again a decrease from 2X to 3X (Table 2). For *Hpa*II digestion, polymorphism percentages were respectively determined as 30.58%, 39.94%, 42.59% and 30.06% for 0, 1X, 2X and 3X ZnO NPs treatments, as 34.39%, 26.61%, 33.83% and 22.97% for 0, 1X, 2X and 3X CuO treatments and as 49.81%, 30.19%, 35.92% and 21.31% for 0, 1X, 2X and 3X γ-Fe_3_O_4_ NPs treatments (Table 2). 

**Figure 2:**
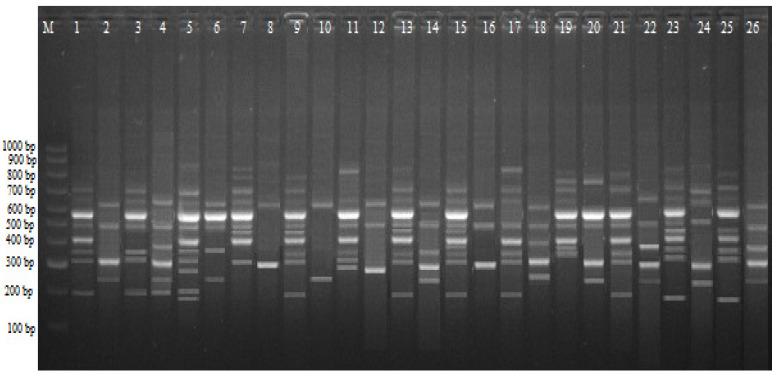
CRED-iPBS profiles for various experimental groups with iPBS 2391 primers in wheat. M, 100–1000 bp DNA ladder; 1, control Msp I; 2, control Hpa II; 3, MS medium supplemented with 0X ZnO NPs Msp I; 4, MS medium supplemented with 0X ZnO NPs Hpa II; 5, MS medium supplemented with 1X ZnO NPs Msp I; 6, MS medium supplemented with 1X ZnO NPs Hpa II; 7, MS medium supplemented with 2X ZnO NPs Msp I; 8, MS medium supplemented with 2X ZnO NPs Hpa II; 9, MS medium supplemented with 3X ZnO NPs Msp I; 10, MS medium supplemented with 3X ZnO NPs Hpa II; 11, MS medium supplemented with 0X CuO NPs Msp I; 12, MS medium supplemented with 0X CuO NPs Hpa II; 13, MS medium supplemented with 1X CuO NPs Msp I; 14, MS medium supplemented with 1X CuO NPs Hpa II; 15, MS medium supplemented with 2X CuO NPs Msp I; 16, MS medium supplemented with 2X CuO NPs Hpa II; 17, MS medium supplemented with 3X CuO NPs Msp I; 18, MS medium supplemented with 3X CuO NPs Hpa II; 19, MS medium supplemented with 0X γ-Fe_3_O_4_ NPs Msp I; 20, MS medium supplemented with 0X γ-Fe_3_O_4_ NPs Hpa II; 21, MS medium supplemented with 1X γ-Fe3O4 NPs Msp I; 22, MS medium supplemented with 1X γ-Fe_3_O_4_ NPs Hpa II; 23, MS medium supplemented with 2X γ-Fe_3_O_4_ NPs Msp I; 24, MS medium supplemented with 2X γ-Fe_3_O_4_ NPs Msp I; 25, MS medium supplemented with 3X γ-Fe_3_O_4_ NPs Hpa II; 26, MS medium supplemented with 3X γ-Fe_3_O_4_ NPs Msp I.

In general, it was found that the non-γ-Fe_3_O_4_ NPs treatment yielded a higher polymorphism percentage in both *Msp*I- and *Hpa*II-digested CRED-iPBS assays (57.96% and 49.81%, respectively). On the contrary, the application of 3X treatments of all nanoparticles decreased both *HpaII* and *MspI* polymorphism percentages. In other words, the 2X treatments of ZnO and CuO NPs had an impact on cytosine methylation status and thus could be classified as hypermethylation when the average polymorphism percentage for *Msp*I digestion was taken into consideration. On the other hand, 3X treatments resulted in an apparent decrease in average polymorphism percentages and impacted methylation status, thus classified as hypomethylation (Table 2 and Figure 2). 

## 3. Discussion

Nanotechnology has improved plant genetics through special nanoparticles (materials with dimensions between 1 and 100 nm), thus enhancing agricultural productivity [27] as growth-promoting nanoparticles may increase yield and quality traits [28]. Compared to bulk materials, nanoparticles of the same material have novel and improved physico-chemical and biological characteristics [29]. According to Nadaroglu et al. [29], nanoparticles at appropriate doses could improve seed germination and plant growth and development, increase yield levels, reduce soil pollution and protect plants against biotic and abiotic stressors. However, there are a limited number of studies on the effects of nanoparticles on induced-DNA methylation, genomic instability and LTR retrotransposon polymorphism. The present findings revealed that different concentrations (0, 1X, 2X and 3X) of ZnO, CuO and γ-Fe_3_O_4_ NPs had a significant role in wheat’s DNA methylation and genomic instability. Micronutrients are essential for plant growth and development; many are involved in catalytic redox reactions. In this study, inter-primer binding site (iPBS) retrotransposon and CRED-iPBS (coupled restriction enzyme digestion-iPBS) techniques were used to define the DNA damage levels and changes in DNA methylation status. As compared to the control treatments, ZnO, CuO and γ-Fe_3_O_4_ NPs treatments increased polymorphism rates and cytosine methylation and decreased GTS values. 

While 1X and 2X ZnO treatments increased polymorphism rate and cytosine methylation, 3X treatments reduced these values compared to the negative control (without Zn). Such opposite effects of ZnO NPs treatments were seen on GTS values. It can be thought that the epigenetic modifications that occur in wheat endosperms exposed to ZnO doses may be related to changes in the expression level of the PI-II gene, which may be a key mechanism responsible for developing plant immunity against stress conditions [30]. A previous study revealed that ZnO nanoparticles similarly affect the transcription factors in wheat and cause a change in the expression level of the HSFA4A gene [31]. Another mechanism of action in the epigenetic polymorphism formed by ZnO nanoparticles in callus tissues may be due to the interaction of zinc with biomolecules and cellular organelles [30]. Zinc is an essential nutrient and plays a vital role in synthesizing chlorophyll, carbohydrates and phytohormones required for plant growth and development [32]. It was reported that zinc nanoparticles enhance the plant growth and yield levels of *Cyamopsis tetragonoloba* L., *Gossypium hirsutum, Lycopercicum esculentum* and *Stevia* [33,34]. It was also reported that, at proper doses, zinc nanoparticles improve shoot and root lengths, chlorophyll and protein contents and increased the yield levels of *Pennistem americanum* L. plants [35]. Excessive quantities of nanoparticles may facilitate oxidative burst, which in turn results in the production of vast quantities of reactive oxygen species (ROS) [36]. It was reported that metal nanoparticles provoke the stress response of plants [37]. According to [38], zinc nanoparticles have significant effects on biomass, root and shoot lengths, chlorophyll and protein contents, as well as enzyme activity in *Cyamopsis tetragonoloba* L. plants. Excessive or deficient micronutrients may facilitate ROS accumulation and disrupt enzyme activity, resulting in cell cycle anomalies and impairing overall biomass, yield and quality [39,40]. 

While 1X CuO treatments increased polymorphism rate and cytosine methylation, 2X treatments reduced them, while 3X treatments increased them compared to the negative control (without Cu). The opposite effects of ZnO treatments were seen on GTS values. It can be thought that the effect of CuO nanoparticles on epigenetic modifications is related to oxidative stress, as shown by studies that analyzed transcriptomic data related to the modification of oxidative-stress-related genes following CuO nanoparticle applications [41]. Copper is essential in electron transport and cell wall metabolism [42] and facilitating ethylene receptors [42]. Copper is an essential nutrient for plants, and since it is involved in various physiological processes, it is used mainly in agricultural activities [43]. For example, copper ions protect plant cells against oxidative damage [44] and facilitate the production of hydroxyl radicals [42] and biochemical pathways [45]. CuO nanoparticles are more soluble and toxic at low concentrations [46]; they can affect the Krebs cycle at sublethal concentrations [42]. It was reported that CuO nanoparticle toxicity negatively affects the seed germination and plant growth of lettuce, mung bean, kidney bean, alfalfa, wheat, chickpea and several other crops [47]. 

The present findings revealed that γ-Fe_3_O_4_ treatments at 1X, 2X and 3X concentrations all reduced polymorphism rate and cytosine methylation compared to the negative control (without Fe). However, an opposite effect was seen on GTS values. It has been reported that iron ions (Fe^2+^) reduce copper absorbance remarkably; therefore, malfunctions may be seen in functions related to the minor copper element in plants. For this reason, it can be thought that iron nanoparticles applied at high doses can regulate many physiological processes that require minor copper elements with epigenetic modifications [48]. Iron (Fe) is an essential nutrient for all organisms. It plays a crucial role in several physiological processes of plants, including chlorophyll biosynthesis, respiration and redox reaction [49]. It was reported that FE nanoparticles decrease chlorophyll content and root hydraulic conductivity, thus influencing nutrient transport within the plant [50]. U Sami and U Rehman [51] indicated that Fe nanoparticles significantly affect the growth and development of *Citrus maxima, Lycopersicum esculentum and Triticum aestivum* plants. FeO nanoparticles promote plant growth and development in soybean [52], wheat [53] and peanuts [54]. Fe nanoparticles improve FRO_2_ gene expression levels, increase ferric reductase activity, enhance iron transformation and improve plant tolerance to iron deficiency [51].

Overall, the present findings revealed that epigenetic changes occurred using nanoparticles instead of the main element in the MS medium. Nanoparticles enhance plant growth and yield when used at proper concentrations. They interact with plant cells and change the biochemical pathway by affecting the regulation of gene expression, which enhances plant growth and development, but they also have inhibitory effects on plant growth and production when used above the optimum levels. Such negative effects could be attributed to nanoparticle type, structure, concentration and application period. Epigenetics encompasses heritable changes in gene functions without directly altering the DNA sequence [55]. Epigenetic mechanisms include DNA methylation and histone modifications [56]. In the present study, non-γ-Fe_3_O_4_ NPs treatment and 2X ZnO and CuO treatments yielded higher polymorphism percentages in both *Msp*I- and *Hpa*II-digested CRED-iPBS assays and thus could be classified as hypermethylation when the average polymorphism percentage for *Msp*I digestion was taken into consideration.

On the contrary, the 3X concentration of all nanoparticles decreased both *HpaII* and *MspI* polymorphisms’ percentages and thus could be classified as hypomethylation. Nanoparticles can influence DNA methylation through two primary mechanisms—the reduced availability of methyl donors and altered activity of DNA methyl-transferases enzymes. The pro-oxidative characteristics of nanoparticles may alter DNA methylation patterns [57]. In addition to oxidative damage, ROS could also alter gene expression levels and thus DNA methylation status [58]. Accordingly, it was previously reported that nanoparticles induce ROS production [59]. Nanoparticles may also result in cytotoxicity, cell death, oxidative stress, genotoxicity and immunotoxicity [60], thus inducing abnormal epigenetic processes [61] and changes in the proteome [62]. Exposure to nanoparticles induces oxidative stress, lipid peroxidation and membrane damage, ultimately resulting in hypomethylation, accompanied mainly by hypermethylation. Environmental factors could also trigger epigenetic changes [46]. In general, it has been observed that nanoparticle structures have an effect on the modification of transcription factors in plants; hence, they are effective both in changing the expression profile and in signal transduction [31]. However, it is known that many factors, such as the size, shape and synthesis method of nanoparticles, can influence epigenetic modifications [63]. In addition, many factors, such as the method of application of nanoparticles on the plant, the dose and the duration of application, can determine the quality and quantity of the effect. Since this process is affected by the changes in many parameters, more studies are needed to clarify the effects of nanoparticles on plants.

## 4. Materials and Methods

The nanoparticles used in the experiment were obtained using the biological reduction method, and their nano size was confirmed as described by Nalci et al [23]. CuO, ZnO and γ-Fe_3_O_4_ nanoparticles were synthesized by green synthesis using a peroxidase enzyme obtained from the Euphorbia amygdaloides plant. Nanoparticles were characterized by SEM, XRD and FTIR analysis. As a result of the characterization findings, it was determined that γ-Fe_3_O_4_ NPs are in the size range of 30–80 nm, ZnO NPs are in the range of 60–80 nm and CuO NPs are in the range of 5–120 nm.

Briefly, iron, copper and zinc elements were removed from the Murashige and Skoog (MS) medium and replaced with a nanoparticle version of these elements at 1X, 2X and 3X concentrations [24]. The embryogenic callus is a green formation with a total plant-forming capacity that develops on callus tissue. After the embryogenic callus developed on the callus, they were transferred to different nutrient media under aseptic conditions to form a whole plant. Genomic DNA was isolated from plantlet leaves obtained from embryogenic callus using the method specified by Hosseinpour et al. [64]. The concentration and quality of genomic DNA were measured with a Nanodrop spectrophotometer (Qiagen Qiaxpert, Qiagen, Hilden, Germany) (Thermo Fisher Scientific, Waltham, MA, USA) and run on 1.5% (*w/v*) agarose gel. Twenty primers were tested for iPBS-PCR amplification [65]. For iPBS analysis, a PCR reaction was carried out by Hosseinpour et al. [22]. Out of 40 iPBS oligonucleotide primers, only 10 resulted in specific and stable DNA profiles in all experimental groups (Table 3). 

CRED-iPBS analysis was conducted as specified by Hosseinpour et al. [64], using the primers listed in Table 3 for amplification. PCR steps were the same as for iPBS analysis described by Hosseinpour et al. [22]. iPBS amplification conditions were: initial denaturation for 3 min at 95 °C; 38 cycles of 15 s at 95 °C, 60 s at 51–56 °C and 60 s at 72 °C and a final extension of 5 min at 72 °C. CRED-iPBS amplification conditions were: an initial denaturation step of 5 min at 95 °C; 42 cycles of 60 s at 94 °C, 60 s at 51–56 °C and 120 s at 72 °C and a final extension step of 15 min at 72 °C. The iPBSs and CRED-iPBS PCR products were separated in a 1.5% agarose gel containing 0.05 µg/mL ethidium bromide, using 1X SB buffer in 100 V for 90 min for electrophoresis. The 100–1000 bp (Sigma Aldrich, MO, USA, No: P1473-1VL) DNA ladder was used to estimate the molecular weight of the fragments. The gels were photographed under UV light in a Universal Hood II (Bio-Rad, Hercules, CA, USA). 

The iPBS and CRED-iPBS banding patterns were analyzed using TotalLab TL120 software (Nonlinear Dynamics Ltd. Newcastle, UK). Polymorphism and genomic template stability (GTS %) were analyzed as described by Hosseinpour et al. [64]. For CRED-iPBS analysis, the average values of polymorphism (%) were calculated for each concentration [23].

## 5. Conclusions

In this study, the effects of different nanoparticles (ZnO, CuO and γ-Fe_3_O_4_) at different concentrations (1X, 2X and 3X) on methylation and epigenetic changes encountered in wheat DNA were investigated. Based on the present findings, it was concluded that nanoparticles play an essential role in DNA methylation and genomic instability. Current findings indicated that epigenetic modification via cytosine methylation could be an essential regulatory mechanism in plants. It was determined that the three metallic nanoparticles, especially at high doses, caused changes in genomic instability. It was concluded that the excess and the absence of minor elements in the wheat plant revealed changes at the nanoscale DNA level. Exposure to nanoparticles can induce epigenetic changes, but the consequences of these changes have not been fully elucidated. Further research is recommended for the high-throughput analysis of genetic and metabolic responses triggered by nanoparticles and to shed light on various aspects of nanoparticle phytotoxicity in plants.

## Figures and Tables

**Table 1:** Molecular sizes (bp) of present/absent bands in iPBS profiles under different nanoparticle treatments.

Primer	* +/− **	Control ***	Experimental Groups											
			ZnO NPs				CuO NPs				γ-Fe_3_O_4_ NPs			
			0 ^a^	1X ^a^	2X ^b^	3X ^c^	0 ^b^	1X ^b^	2X ^b^	3X ^b^	0 ^c^	1X ^c^	2X ^c^	3X ^c^
iPBS 2382	+	9	627; 166	239	850; 520	-	458	-	560; 265	-	625; 300	580	750; 536	625; 245
	−		605	287	485	698	308	146	335	420	352	-	485; 146	335
iPBS 2383	+	6	430; 307	440; 207	520	330; 207	-	-	610	327	188	350	-	218
	−		-	350	400; 258	350	515; 285	400	258	515; 400	651	285	651	400
iPBS 2384	+	7	608	670; 505	682; 426	285	300	300	483; 322	584	480; 265	185	465	-
	−		-	359	315; 105	-	655; 250	-	-	359	450	550; 450	315; 105	655
iPBS 2385	+	8	397	-	100	274	350	-	-	428; 125	385	-	-	505
	−		-	305; 158	571; 409	-	-	550; 350	675	-	450; 158	158	-	-
iPBS 2386	+	6	524	440	350; 205	450	287	405	620	355	-	-	450; 366	-
	−		352	-	600	-	-	750	450	-	450; 305;205	450; 205	600	-
iPBS 2387	+	5	650	392	-	450; 205	-	650; 258	715	115	320	-	452	502
	−		560; 367	-	367; 239	600	239	-	-	367	560; 153	239	285	153
iPBS 2388	+	6	-	342	467	565; 295	487	378	350; 258	405	-	482	489; 326	-
	−		221	285	362	160	160	-	221; 160	-	450	650; 362	285	362
iPBS 2389	+	8	118	-	404	-	858; 465	157	248	-	580	571	535; 228	776
	−		-	450	-	285	550	360	-	517	600; 350; 140	-	-	-
iPBS 2390	+	7	657	267	-	-	-	850; 609	615	-	305; 127	422	-	115
	−		632; 362	-	605	495	362	405	-	-	-	-	605	503
iPBS 2391	+	5	547	620; 552	-	507	-	535	310; 539	618; 250	778	-	-	250
	−		-	450	350	350	650; 407	-	350	-	-	209; 450	-	-
Total band		67	17	19	23	17	18	16	20	15	25	17	19	14
Polymorphism (%)			25.37	28.35	34.32	25.37	26.86	23.88	29.85	22.38	37.31	25.37	28.35	20.89
GTS value			74.62	71.64	65.67	74.62	73.13	76.11	70.14	77.61	62.68	74.62	71.64	79.10

*, ** and ***, appearance of a new band, disappearance of a normal band and without hormone, respectively; 0 ^a^, MS medium containing 0X ZnO NPs; 1X ^a^, MS medium containing 1X ZnO NPs; 2X ^a^, MS medium containing 2X ZnO NPs; 3X ^a^, MS medium containing 3X ZnO NPs; 0 ^b^, MS medium containing 0X CuO NPs; 1X ^b^, MS medium containing 1X CuO NPs; 2X ^b^, MS medium containing 2X CuO NPs; 3X ^b^, MS medium containing 3X CuO NPs; 0 ^c^, MS medium containing 0X γ-Fe_3_O_4_ NPs; 1X ^c^, MS medium containing 1X γ-Fe_3_O_4_ NPs; 2X ^c^, MS medium containing 2X γ-Fe_3_O_4_ NPs; 3X ^c^, MS medium containing 3X γ-Fe_3_O_4_ NPs.

**Table 2:** Results of CRED-iPBS analysis; the molecular size of bands and polymorphism percentages.

Primer	M */H **	+ ***/− ****	Control *****	Experimental Groups											
				ZnO NPs				CuO NPs				γ-Fe_3_O_4_ NPs			
				0 ^a^	1X ^a^	2X ^b^	3X ^c^	0 ^b^	1X ^b^	2X ^b^	3X ^b^	0 ^c^	1X ^c^	2X ^c^	3X ^c^
iPBS 2382	M	+	8	658; 434	420; 156	590;	660	645	450	660; 450; 305	** - **	785; 475; 325; 120	** - **	650; 450	-
		−		-	600	250; 165	-	-	250	** - **	485; 375	298	205	165	345
	H	+	6	575	497	320; 139	365	335	150; 216	685; 455		** - **	168; 102	605	335
		−		415	305	-	-	180	-	-	350	254; 128	-	128	-
iPBS 2383	M	+	5	754; 515	555;450	550; 362; 125	538	360	780	785; 650	375	** - **	605; 515	785	645; 360
		−		-	245	405; 350	-	** - **	** - **	** - **	-	350; 245; 165	-	525	-
	H	+	4	-	450; 325	667; 240	-	455; 300	409; 125	600	-	480	170	-	-
		−		405	-	-	405	** - **	** - **	395	540	305	155	305; 155	305
iPBS 2384	M	+	7	-	604; 425	475; 282	378	655; 509; 430	** - **	708; 506	** - **	360; 270; 235	** - **	505; 337; 125	475; 345
		−		390; 240	450	350	-	** - **	625; 120	** - **	390	350; 205	450; 120	** - **	-
	H	+	6	285	-	125	465	495	** - **	** - **	275	760; 605; 375	260	550	262
		−		-	350	425; 350	-	250	165	450	450	165	568; 425	425; 350	-
iPBS 2385	M	+	11	450	808; 745; 625	350; 128	825; 486	** - **	760; 456	785; 345	625	458; 325; 124	** - **	850; 650; 485; 120	625
		−		340	-	785	-	325; 410	325	440	** - **	705; 650	605	-	-
	H	+	10	-	695	374; 125	560; 393	260	** - **	755; 550; 469	745	710; 650	330; 280	355	-
		−		425	525; 365	-	-	300; 170	751; 636, 225	** - **	225	345	** - **	450; 345	525
iPBS 2386	M	+	4	340; 115	-	405; 300	807; 485	300; 220	-	615; 430	405	345; 262	600	565; 450	475
		−		-	255; 458		654	-	458; 355	355	** - **	-	255	-	-
	H	+	8	-	-	505; 118	715	450	650	685	** - **	600; 450	605	655	305
		−		525	400; 225	150	605; 150	360; 320	** - **	** - **	225; 150	375; 225		400; 320	-
iPBS 2387	M	+	3	285	-	605	-	425	785; 250	** - **	-	680; 510	670; 466	485	689
		−		185	185	-	650; 580	580	-	650; 185	580; 185	** - **	580	650	-
	H	+	9	-	420;285	605; 225	450	500	** - **	550	780	450; 245;125	** - **	885; 745; 600	-
		−		850; 405; 365	405;160	-	715	125	365	365; 125	160	345; 205	125	** - **	550; 205
iPBS 2388	M	+	7	450; 226	250; 115	510; 258	388	520; 450	487	650; 255; 125	425	360; 220	** - **	655; 505	650
		−		-	-	405; 100	-	** - **	** - **	** - **	** - **	185; 100	575; 345	405	** - **
	H	+	8	250	397; 285	440; 338; 112	746; 350; 119	380	455; 350	580; 375; 120	** - **	498	624	404; 348	-
		−		535; 453	308	-	-	-	** - **	405	355; 105	500; 462	-	-	500
iPBS 2389	M	+	11	375; 198	785; 625; 320	625; 405	550	680; 465; 345	460; 128	645; 460; 355	** - **	605; 485; 325	652	496; 320	128
		−		-	490	-		** - **	** - **	** - **	105	447	-		520
	H	+	8	285; 159	153	750; 418	-	785; 560; 245	** - **	424; 335	** - **	450; 355; 265	600; 505; 485	525	** - **
		−		255	350; 228	402; 350	228;125	450	350	320	350	255; 125	-	350	320
iPBS 2390	M	+	5	490; 350	485; 309	365; 263	785; 460; 340	** - **	750	464; 128	** - **	560; 258	365	775	-
		−		-	180	450; 180	-	654; 350	** - **	450	350; 180	350;180	-	** - **	245
	H	+	4	365	-	582; 278; 145	525;410	669; 285	680; 490	325	** - **	458	635	-	605
		−		125	600;450	-		** - **	-	125	600	345	125	600; 450	125
iPBS 2391	M	+	8	-	850; 290;185	758; 800; 450	750; 450	800; 285	810; 450	456; 378	830	805; 753; 385	800	850; 450	750; 330
		−		400	-	350; 200	-	705; 200	** - **	** - **	555	315; 200	645	** - **	495
	H	+	4	350; 200	550; 360	-		** - **	346	** - **	345	775; 555	390	650	350
		−		-	300	490; 245	490; 300	245	** - **	245	-	605	** - **	-	-
Polymorphism %	M			32.77	41.34	50.88	31.50	35.60	29.50	43.01	24.84	57.96	32.28	37.94	23.85
	H			30.58	39.94	42.56	30.06	34.39	26.61	33.83	22.97	49.81	30.19	35.92	21.31

*, *, ***, **** and *****, M—Msp I, H—Hpa II, appearance of a new band, disappearance of a normal band and without hormone, respectively. 0 ^a^, MS medium containing 0X ZnO NPs; 1X ^a^, MS medium containing 1X ZnO NPs; 2X ^a^, MS medium containing 2X ZnO NPs; 3X ^a^, MS medium containing 3X ZnO NPs; 0 ^b^, MS medium containing 0X CuO NPs; 1X ^b^, MS medium containing 1X CuO NPs; 2X ^b^, MS medium containing 2X CuO NPs; 3X ^b^, MS medium containing 3X CuO NPs; 0 ^c^, MS medium containing 0X γ-Fe_3_O_4_ NPs; 1X ^c^, MS medium containing 1X γ-Fe_3_O_4_ NPs; 2X ^c^, MS medium containing 2X γ-Fe_3_O_4_ NPs; 3X ^c^, MS medium containing 3X γ-Fe_3_O_4_ NPs.

**Table 3:** Reactive primers used in iPBS PCR and their annealing (Ta) temperatures.

No	Primer Name	Sequence (5′ to 3′)	Tm (°C)	CG (%)
1	iPBS 2382	TGTTGGCTTCCA	44.9	50
2	iPBS 2383	GCATGGCCTCCA	50.5	66.7
3	iPBS 2384	GTAATGGGTCCA	40.9	50
4	iPBS 2385	CCATTGGGTCCA	45.7	58.3
5	iPBS 2386	CTGATCAACCCA	41.4	50
6	iPBS 2387	GCGCAATACCCA	47.3	58.3
7	iPBS 2388	TTGGAAGACCCA	43.4	50
8	iPBS 2389	ACATCCTTCCCA	43	50
9	iPBS 2390	GCAACAACCCCA	47.6	58.3
10	iPBS 2391	ATCTGTCAGCCA	43.6	50

## Data Availability

Data are contained within the article.
